# Ultrasound guidance reduces pneumothorax rate and improves safety of thoracentesis in malignant pleural effusion: report on 445 consecutive patients with advanced cancer

**DOI:** 10.1186/1477-7819-12-139

**Published:** 2014-05-02

**Authors:** Luigi Cavanna, Patrizia Mordenti, Raffaella Bertè, Maria Angela Palladino, Claudia Biasini, Elisa Anselmi, Pietro Seghini, Stefano Vecchia, Giuseppe Civardi, Camilla Di Nunzio

**Affiliations:** 1 Oncology-Hematology Department, Piacenza Hospital, Via Taverna, 49, 29121 Piacenza, Italy; 2 Unit of Biostatistics and Epidemiology, Piacenza Hospital, Via Taverna, 49, 29121 Piacenza, Italy; 3 Laboratory of Cancer Chemotherapy Unit (UFA), Piacenza Hospital, Via Taverna, 49, 29121 Piacenza, Italy; 4 Internal Medicine, Fiorenzuola Hospital, 29017 Fiorenzuola D’Arda, Italy

**Keywords:** Ultrasound, Pneumothorax, Thoracentesis, Malignant pleural effusion, Safety, Cancer patients

## Abstract

**Background:**

Malignant pleural effusion (MPE) is an extremely common problem affecting cancer patients, and thoracentesis is an essential procedure in an attempt to delineate the etiology of the fluid collections and to relieve symptoms in affected patients. One of the most common complications of thoracentesis is pneumothorax, which has been reported to occur in 20% to 39% of thoracenteses, with 15% to 50% of patients with pneumothorax requiring tube thoracostomy.

The present study was carried out to assess whether thoracenteses in cancer patients performed with ultrasound (US) guidance are associated with a lower rates of pneumothorax and tube thoracostomy than those performed without US guidance.

**Methods:**

A total of 445 patients were recruited in this retrospective study. The medical records of 445 consecutive patients with cancer and MPE evaluable for this study, undergoing thoracentesis at the Oncology-Hematology and Internal Medicine Departments, Piacenza Hospital (Italy) were reviewed.

**Results:**

From January 2005 to December 2011, in 310 patients (69.66%) thoracentesis was performed with US guidance and in 135 (30.34%) without it. On post-thoracentesis imaging performed in all these cases, 15 pneumothoraces (3.37%) were found; three of them (20%) required tube thoracostomy. Pneumothorax occurred in three out of 310 procedures (0.97%) performed with US guidance and in 12 of 135 procedures (8.89%) performed without it (*P* <0.0001). It must be emphasized that in all three patients with pneumothorax requiring tube thoracostomy, thoracentesis was performed without US guidance.

**Conclusions:**

The routine use of US guidance during thoracentesis drastically reduces the rate of pneumothorax and tube thoracostomy in oncological patients, thus improving safety as demonstrated in this study.

## Background

Malignant pleural effusions (MPE) are an extremely common problem affecting cancer patients both at the onset or during the course of the disease, and thoracentesis is a commonly performed procedure whereby pleural fluid is aspirated percutaneously through the chest wall for diagnostic or therapeutic purpose.

Potential complications of thoracentesis are pain (5%), cough (24%), shortness of breath (15%), vasovagal reaction (3%), and pneumothorax (20% to 39%) [1].

Less common complications of thoracentesis include inadvertent liver or splenic puncture, bleeding, subcutaneous emphysema, re-expansion pulmonary edema, and infection [1]. Iatrogenic pneumothorax resulting from thoracentesis increases morbility, mortality, and length of hospitalization. In addition, chest tube insertion may be required in up to 50% of cases, with a mean duration of placement of approximately 4 days [2,3]. Approximately 1.5 million people are found to have pleural effusion each year in the United States [4], and although thoracentesis is typically considered by physicians a relatively safe procedure with few complications [4], the incidence of pneumothorax has been reported to be as high as 20% to 39% [5].

Factors that have been shown to reduce the rate of pneumothorax include the performance by experienced personnel [6], as well as the use of US guidance [5,7-9]. While the usefulness of US guidance in reducing the incidence of pneumothorax from thoracentesis has been reported in several studies [5,7-9] and in a recent meta analysis [10], to our knowledge the incidence of pneumothorax from US-guided or unguided bedside thoracentesis in cancer patients is less well documented.

The purpose of this study was to assess the rate of pneumothorax associated with US-guided or unguided thoracentesis in clinical practice in cancer patients attending the oncological-hematological and internal medicine departments of a North Italian general hospital, the Piacenza Hospital.

## Methods

After approval by the institutional review board (Ethic Committee Azienda Sanitaria di Piacenza (Italy)), a search was conducted in the medical records of cancer patients to identify those patients with pleural effusion who underwent thoracentesis.

A total of 462 consecutive patients with cancer who underwent thoracentesis at the oncology-hematology and internal medicine departments over a 6-year period (2 January 2005 to 31 December 2011) were identified.

The medical records of six of these patients were incomplete and they were excluded from the study.

The following clinical data were abstracted from the medical records: clinical setting (inpatient and outpatient); age; diagnosis; sex; indications as to thoracenthesis (diagnostic or therapeutic, diagnostic, and therapeutic); chest radiography findings pre-and post- thoracentesis; pneumothorax; and tube thoracostomy rate.

Eleven patients in whom no prior chest X-ray or following thoracentesis were available or with pre-existing hydropneumothorax at admission to our departments were excluded from the study.

The final group of cases which was the object of this study consisted of 445 evaluable patients.

The patients were divided into two groups: group A, conventional thoracentesis traditional land mark method); and group B, US-guided thoracentesis.

US-guided or conventional thoracentesis was performed by one of five experienced physicians of the departments (LC, PM, RB, MAP, GC) both for groups A and B.

The decision to perform US-guided or conventional thoracentesis was made by the availability or otherwise of US machines at bedside at the moment of thoracentesis. The two groups were not homogeneous since in our departments thoracentesis is performed preferably with US guidance; diagnostic thoracenteses were 5.19% in group A and 11.29% in group B.

All the patients in this study had a pre- and post-thoracentesis chest X-ray.

Once the location of the pleural fluid was identified by physical examination or by US (Esaote, Genoa, Sonosite, Milan, Italy equipped with two transducers between 3.5 to 7.5 MHZ), the area was cleaned with povidone-iodine and local anesthesia and (1% lidocaine without epinephrine) was injected intradermally subcutaneously using a 22-gauge needle into the parietal pleura with or without US guidance. A 14-gauge cannula catheter needle system (ABBOCATH.T, HOSPIRA Inc., Philippines) was used to enter the pleural space at the same point as the local anesthesia, with or without US guidance.

Once the pleural space was entered, the needle was withdrawn and fluid was removed through the catheter. All patients included in the study signed informed consent before undergoing the procedure. This study was approved by the local ethical committee.

### Statistical analysis

Categorical variables were summarized as counts and percentages, continuous variables using median and range.

Comparisons of group A *versus* group B, pneumothorax *versus* no pneumothorax, outpatients *versus* inpatient for various variables were performed using Yates corrected chi square test. In all cases, two-tailed *P* values of 0.05 or less were considered statistically significant. All data were analyzed with OPENEPI statistical computer software. When at least one expected value (row total * column total/grand total) was <5, Fisher’s exact test was used. Student’s *t*-test to calculate the difference between the two groups and the standard deviation was used.

## Results

Clinical characteristics of the 445 patients who underwent US-guided or unguided thoracentesis are reported in Table 1.

**Table 1:** Clinical characteristics of 445 cancer patients with malignant pleural effusion undergoing US-guided (group B) and conventional thoracentesis (group A)

	**Group A**		**Group B**		***P *** **value**
	** N**		** N**		
	135	30.34%	310	69.66%	
Inpatient/Outpatient (N)	72/63			84/226	0.0001
Male/Female (N)	75/60		177/133		0.843
Age (mean) (range)	70.9 (19-87)		71 (18-88)		0.916
Final diagnosis	N (%)				
Lung cancer	70 (51.86)		125 (40.32)		0.031
Breast cancer	56 (41.48)		119 (38.39)		0.610
Gastrointestinal cancer	4 (2.96)		31 (10)		0.019
Other	5 (3.70)		35 (11.29)		0.017
Diagnostic thoracentesis	7 (5.19)		35 (11.29)		0.064
Therapeutic thoracentesis	103 (76.30)		199 (64.19)		0.016
Diagnostic and therapeutic thoracentesis	25 (18.51)		76 (24.52)		0.206
Volume of pleural fluid removed (mL)					
Median (range)	750 40-1,700		1100 70-2,300		0.447

All 445 cases had malignant pleural effusion.

There were 252 male (56.63%) and 193 female (43.37%) patients. The mean age of the group was 70.96 (±9.215) years (range, 18-88 years), 156 (35.06%) were inpatients: 72 in group A and 84 in group B, and 289 (64.94%) outpatients, 63 in group A and 226 in group B.

A total of 195 patients had lung cancer (43.82%), 175 breast cancer (39.33%), 35 gastrointestinal cancer (7.86%), 40 (8.99%) other cancers (mesotelioma, lymphoma, gynaecological cancer, and so on). US was used to guide thoracentesis in 310 of 445 cases (69.66%), including 37 out of 42 (88.1%) diagnostic located thoracentesis, while in 135 (30.34%) cases thoracentesis was performed without US guidance.

The volume of fluid removed was higher for thoracentesis performed with US guidance compared with the procedures done without US guidance: median; 1,100 mL (range, 70-2,300 mL) *versus* 750 mL (range, 40-1,700 mL) (Table 1). There were no significant correlations between the drainage amount and pneumothorax (*P* value = 0.447).

Fifteen of 445 patients (3.37%) who underwent thoracentesis had pneumothorax; among them, three patients (20%) required insertion of a chest tube for a symptomatic or enlarging pneumothorax (Table 2).

**Table 2:** Number of pneumothorax and chest tube insertion following conventional thoracentesis and US-guided thoracentesis

**Pneumothorax and chest tube insertion in cancer patients undergoing thoracentesis**			**Conventional thoracentesis**		**Thoracentesis with US guidance**		***P *** **value**
	**N**	**%**	**N**	**%**	**N**	**%**	
All patients	445	100	135	30.34	310	69.66	
Pneumothorax	15	3.37	12	8.89	3	0.97	0.00001
Chest tube insertion	3	20	3	2.22	0	0	0.054

In group A (conventional thoracentesis) 12 pneumothoraces occurred (8.89%), while in group B (thoracentesis performed with US guidance) only three (0.97%) pneumothoraces occurred (*P* <0.0001) (Table 2).

All three chest tubes were inserted in patients with pneumothorax secondary to thoracentesis performed without US guidance. No significant correlations were found between the occurrence of pneumothorax after thoracentesis and type of cancer.

## Discussion

Thoracentesis is a diagnostic and therapeutic procedure that is routinely performed for evaluation of pleural effusion and to relieve symptoms.

The most common reported thoracentesis complication is the puncture of the visceral pleura, which can cause a pneumothorax [10].

US guidance allows the physician to determine a more accurate needle insertion depth into the intercostal space and thus reduces the incidence of pneumothorax [2,3,5].

It must be emphasized that most studies of US-guided thoracentesis do not use real-time guidance for needle insertion, but insert the needle immediately after identification, with US, of the appropriate site [4].

In our study, the needle was inserted under US real-time guidance, allowing a procedure which was safer and gave less discomfort to the patient. In fact, US guidance also allows for dynamic needle visualization, enabling precise needle placement.

Thoracentesis is typically thought to be a relatively safe procedure with few complications; the incidence of pneumothorax, however, has been reported to be as high as 20% to 39% [5].

The National Confidential Enquiry into perioperative deaths has reported one death resulting from a procedural-induced pneumothorax [11].

It must be emphasized that less serious discomfort (though unpleasant for the patient), clinicians’ time, hospital stay, and economic costs are variables rates from iatrogenic pnemothorax [12].

Several studies have demonstrated that US guidance can reduce the rate of pnemothorax [5,7-9]. Grogan et al. [5] found a significant reduction of the pneumothorax rate when US was utilized for identification of needle placement (0% *versus* approximately 29%).

Similar reductions in the rate of pneumothorax were reported by other authors [7-9].

In addition the occurrence of pneumothorax requiring tube thoracostomy is also significantly reduced with US guidance as reported in the present study, and this is considered even more clinically relevant [8,9].

Another very important benefit of US guidance is the increased success in thoracentesis also after a clinically failed directed thoracentesis: it has been demonstrated that fluid can be successfully obtained in up to 88% of patients after an unsuccessful by clinically guided thoracentesis [9,13-16] and even more importantly in 58% of clinically attempted ‘dry taps’ the needle insertion site was found to be below the diaphragm [15].

US guidance increased the rate of accurate site selection by 26% and decreased the number of mean misses, the number of potentially dangerous needle insertion sites by 10% when compared to fluid localization by physical examination and chest radiography [16].

In our study, we found a statistical significant reduction in the pneumothorax rate after thoracentesis when US guidance was utilized: three (0.97%) *versus* 12 (8.89%) without US guidance (*P* <0.0001), and also a reduction in tube thoracostomy: 0 (0%) *versus* three (100%).

It must be emphasized that our data concern cancer patients. To our knowledge, this is the first report of this setting of patients.

In view of the above, the advantages of US-guided thoracentesis are demonstrated also in cancer patients; and we recommend that the use of US for thoracentesis be considered for all patients with cancer and pleural effusion. Should patients undergoing thoracentesis be referred to interventional radiologists for US-guided procedure, or should it be a clinician (oncologist, hematologist, internist, or a physician from another medical specialty) that performs the US-guided procedure? Thoracentesis by interventional radiologists under US guidance include the necessity of moving the patient to the radiology suite with the additional cost of the procedure in the US suite [1]. Although interventional US guidance is well-established in such specialties as radiology, cardiology, and obstetrics and gynecology, it is becoming more common across a range of specialties and procedures [17].

In addition, it must be emphasized that the use of US is not limited to radiologists [4]; the American Medical Association policy favors US imaging technique diffusion in medical practice [18]; the American College of Emergency Physicians and the American College of Surgeons support the use of US by members of their societies and address ways to obtain and maintain competence as well as ensuring quality control [19,20]. We agree with previous report [4] that US is an excellent teaching tool, and in our departments we use it routinely to reinforce the physical examination of residents and medical students.

In our departments oncologists, hematologists, and internists have performed US imaging procedures as well as interventional US in clinical practice for the management of patients (diagnosis, staging, restaging follow-up) for almost 25 years [12,21-26]. So this procedure was early applied to guide thoracentesis, performed by physicians at the bedside without moving the patients to the radiology suite.

David Kopman Feller 2007 [27] stated that US is an easily learned technique that not only enhances the physical examination but has also the distinct advantage of being a portable tool that can provide real-time guidance for thoracentesis and other interventional procedures such as biopsy, abscess drainage, paracentesis, and central venous catheter insertion. In addition, this procedure can be performed easily at the bedside as reported in our study.

Gordon et al. [10] in their systematic review and meta-analysis showed that the most important strategy to reduce pneumothorax rates following thoracentesis, was the use of US guidance, and they encourage institutions to consider a policy of uniform use of US guidance for thoracentesis, and more recently Patel et al. [17] showed that US-guided thoracentesis is associated with lower total hospital stay, lower costs, and lower incidence of pneumothorax and hemorrhage. In addition, the Patient Protection and Affordable Care Act of 2010 and the Centers for Medicare and Medicaid Services Reporting Hospital Quality Data for Annual Payment Update Program Quality Measures FY 2012–2014 identify as major goals for better healthcare delivery, safer medical care, and avoidance of preventable complications, including iatrogenic pneumothorax [28-30].

More recent literature highlights the safety and efficacy of US-guided thoracentesis. These data indicate that US guidance is associated with decreased risk of pneumothorax. This complication results in a measurable increase in hospitalization costs and length of stay [31,32].

## Conclusions

The results of the present study, show that US-guided thoracentesis in cancer patients is a safe, cheap, and effective technique in reducing both pneumothotax rate and tube thoracostomy rate.

Based on these findings, on previous reports [1-10,17,31] and on the availability of portable US machines, we believe that the time has come to perform all thoracenteses in cancer patients under US guidance, thus improving safety and avoiding complications and patient discomfort.

## Competing interests

The authors have no conflicts of interest or financial support to declare.

## Authors’ contributions

LC and PM designed the research, LC, PM, RB, MAP, CB, EA, PS, SV, GC and CDN contributed to acquisition of data, LC, PS, SV and CDN analyzed and interpreted data. All authors read and approved the final manuscript.
